# BMC Surgery reviewer acknowledgement, 2012

**DOI:** 10.1186/1471-2482-13-6

**Published:** 2013-03-20

**Authors:** Thomas A Rowles

**Affiliations:** 1 BioMed Central, Floor 6, 236 Gray's Inn Road, London, WC1X 8HB, United Kingdom

## Abstract

**Contributing reviewers:**

The editors of BMC Surgery would like to thank all our reviewers who have contributed to the journal in Volume 12 (2012).

## 

Roland Andersson

Sweden

Vanni Agnoletti

Italy

Usama Ahmed Ali

Netherlands

Luca Ansaloni

Italy

Ojan Assadian

Austria

Gian Luca Baiocchi

Italy

Savio Barreto

India

Kathryn Beauchamp

USA

Susanne Beckebaum

Germany

Aneel Bhangu

United Kingdom

Fausto Biancari

Finland

Nikolai Bildzukewicz

USA

Richard Bold

USA

Brandon Broome

USA

Steven Brown

United Kingdom

Walter Brunner

Switzerland

Raymond Cartier

Canada

Javier Castroagudin

Spain

Roberto Cirocchi

Italy

Mechteld De Jong

Netherlands

Maria Debora De Pasquale

Italy

Ishan De Zoysa

Sri Lanka

Keith Delman

USA

Franklin Dexter

USA

Federico Di Rocco

France

Werner Draaisma

Netherlands

Traian Dumitrascu

Romania

Aria Fallah

Canada

Eriberto Farinella

Italy

Stefan Farkas

Germany

Mariano Fernandez-Fairen

Spain

Florian Fitzal

Austria

Jason B Fleming

USA

Michael Flierl

USA

Jens Peter Garne

Denmark

Cengiz Gebitekin

Turkey

Clemens Gerstenkorn

Germany

Helene Gilgenkrantz

France

Vincent Gremeaux

France

Jochen Grommes

Germany

Raquel Gutierrez-Gonzalez

Spain

Anna Hambraeus

Sweden

Werner Hartwig

Germany

Ali Can Hatemi

Turkey

Beate Herbig

Germany

Min-Wei Huang

Taiwan

Hiroo Imazu

Japan

Kshama Jaiswal

USA

Hiromitsu Jinno

Japan

Christian Jurowich

Germany

Nicolas Kairinos

South Africa

Chang Moo Kang

South Korea

Lewis Kaplan

USA

Wolfram Karenovics

Switzerland

Georgios Kasimatis

Greece

Tobias Keck

Germany

Dezső Kelemen

Hungary

G. Edward Kimm Jr

USA

Olivia Kituuka

Uganda

Akira Kobayashi

Japan

Manousos Konstadoulakis

Greece

Gregory Kouraklis

Greece

Simon Kuesters

Germany

Hakan Kulacoglu

Turkey

Kuno Lehmann

Switzerland

Yan Li

China

Bennie Lindeque

USA

Meryl Livermore

USA

Alex Macario

USA

Norman Machado

Oman

Roberto Manfredi

Italy

Ehsan Mansourian

Iran

Daniele Marrelli

Italy

Cyril Mauffrey

USA

Tommaso Mineo

Italy

Massimiliano Mistrangelo

Italy

Rohin Mittal

India

Luis Molfetta

France

Yvonne Mondorf

Germany

Umberto Morelli

France

Mohammad Kazem Moslemi

Iran

Nicolas Murith

Switzerland

Noel Naidoo

South Africa

Atsushi Nanashima

Japan

Gianantonio Nappi

Italy

Julia Neily

USA

Stephane Noble

Switzerland

Robert Obermaier

Germany

Margaret Olsen

USA

Carlos Ordonez

Colombia

Daniel Orringer

USA

Jose Maria Pascual

Spain

Cesira Pasquarella

Italy

Douglas Paull

USA

Frank Pfeffer

Norway

Fredric Pieracci

USA

Elia Poiasina

Italy

Biju Pottakkat

India

Julie Reeve

New Zealand

Burckhardt Ringe

Tunisia

Daniel Rittirsch

Switzerland

Gabriel Rodrigues

India

Geert Roeyen

Belgium

Marco Roffi

Switzerland

David Rudnick

USA

Thibault Sablon

Belgium

Reza Saidi

USA

Milic Sandra

Croatia

Thomas Scalea

USA

Hubert Scheidbach

Germany

Peter Schemmer

Germany

Badri Man Shrestha

United Kingdom

Constantine Spanos

Greece

Philip Stahel

USA

Oliver Strobel

Germany

Frederich Syffold

France

Konstantinos Tepetes

Greece

Wolfgang Thasler

Germany

Oliver Thomusch

Germany

Christine Toevs

USA

Christian Toso

Switzerland

Parsia Vagefi

USA

Cees Van Der Schans

Netherlands

Aude Vanlander

Belgium

Burkhard Von Rahden

Germany

Sebastian Weckbach

USA

Ulrich Friedrich Wellner

Germany

Uwe Wittel

Germany

Gerhard Wolf

Austria

Hiroki Yamaue

Japan

Nicola Zampieri

Italy

## Pre-publication history

The pre-publication history for this paper can be accessed here:

http://www.biomedcentral.com/1471-2482/13/6/prepub

